# Evaluation of the analytical performance of the PC100 platelet counter

**DOI:** 10.1186/s12959-021-00283-w

**Published:** 2021-05-04

**Authors:** Magdolna Nagy, Sepanta Fazaeli, René van Oerle, Hugo ten Cate, Marcel Schemmann, John Sherry, Gillian Kelleher, Henri M. H. Spronk

**Affiliations:** 1 Departments of Internal Medicine and Biochemistry, Cardiovascular Research Institute Maastricht (CARIM), Maastricht University Medical Centre+, Universiteitssingel 50, Maastricht, 6229 ER The Netherlands; 2 2M Engineering, Valkenswaard, The Netherlands; 3 FOCE Technology International BV, Maria Hoop, The Netherlands

**Keywords:** Platelet counter, Hematology analyzer, Point-of-care device, Method comparison

## Abstract

**Introduction:**

Platelet count can be altered in various diseases and treatments and measuring it may provide better insight into the expected outcome. So far, quantification of platelet count is done within laboratory conditions by using established hematology analyzers, whereas a point-of-care device could be used for this purpose outside of the clinical laboratories.

**Aim:**

Our aim was to assess the closeness of agreement between a newly developed point-of-care PC100 platelet counter and two reference methods (Sysmex® XP-300, Sysmex® XN-9000) in measuring platelet counts in whole blood and platelet-rich-plasma (PRP).

**Method:**

Whole blood was obtained from 119 individuals, of which 74 were used to prepare PRP samples. Whole blood platelet count was measured by the two reference methods and the PC100 platelet counter. PRP was prepared from the whole blood and platelet count was adjusted to the range of 250–3600 × 10^3^/μl and measured with the PC100 platelet counter and Sysmex® XP-300.

**Results:**

A median difference of − 1.35% and − 2.98% occurred in whole blood platelet count between the PC100 platelet counter and the Sysmex® XP-300 and Sysmex® XN-9000, respectively. A strong linear correlation (r ≥ 0.98) was seen in both cases and regression equations indicated neither a constant nor a proportional bias between the methods. Direct comparison of the two reference methods revealed a median difference of − 1.15% and a strongly linear relationship (*r* = 0.99). Platelet count in PRP resulted in a median difference of 1.42% between the PC100 platelet counter and the reference method, Sysmex® XP-300. While the difference between two methods increased with concentration of platelets in PRP, a strong linear relationship remained throughout the whole measuring interval indicated by the high correlation coefficient (*r* = 0.99). Assessment of the predicted bias at predefined platelet counts showed that the bias in platelet counts falls within the acceptance criterion for both whole blood and PRP measurements.

**Conclusions:**

Our results show that the PC100 platelet counter can be used interchangeably with the reference methods for determining platelet counts.

## Introduction

The precise assessment of the platelet count in whole blood is crucial in clinical hematology as low platelet count may lead to bleeding complications. The normal platelet count is widely considered to be 150-400 × 10^3^/μl in whole blood and its aberrations have been associated not only with hemostatic and thrombotic diseases but also with cancer and chronic inflammatory diseases [1–3]. Overall, the accumulated data suggests that platelet count might be a possible biomarker or prognostic marker for various diseases (e.g., colorectal cancer, vasculitis, viral infections, etc.).

Currently, there are various automated methods available for determination of platelet counts, with the majority mainly performed in controlled laboratory conditions [4, 5]. Moreover, several point-of-care (POC) hematology analyzers have been tested in research and clinical settings providing complete blood cell (CBC) measurements including platelet counts [6–8]. However, these hematology analyzers are focused on whole blood samples, and not on platelet rich plasma (PRP) samples which may contain an extreme high platelet count. The capability for quantifying high platelet counts is relevant in the light of regenerative medicine where PRP has been proposed as a promising candidate for treatments [9, 10]. In regenerative medicine, PRP containing a high number of platelets (> 900 × 10^3^/μl) can be used [11], and hence a reliable platelet counter with the capability of measuring such extreme platelet counts would be favorable.

The PC100 automated platelet counter has been recently developed with the intended use as a point-of-care device outside of laboratories for measuring platelet count at both the lower and higher ranges. The PC100 platelet counter is an optical platelet counter using a patented optical technique that enables the precise quantification of platelets, even at an extremely high concentration [12].

The aim of this study was to assess the closeness of agreement between the PC100 platelet counter and two reference methods, Sysmex® XP-300 and Sysmex® XN-9000 for determining platelet counts in whole blood and PRP.

## Methods

### Blood collection and preparation

Venous blood was collected from 119 volunteers after informed consent was obtained in accordance with the Declaration of Helsinki. Blood was collected on K_2_-EDTA (Vacuette®, Greiner Bio, Kremsmünster, Austria) for platelet count determination in whole blood. All measurements were performed within 3 h after blood collection to ensure platelet count stability [13].

Furthermore, whole blood from 74 out of 119 volunteers was also collected on 1:6 (v/v) acid citrate dextrose (ACDA; Vacuette®, Greiner Bio, Kremsmünster, Austria) and 3.2%(w/v) trisodium citrate (Vacuette®, Greiner Bio, Kremsmünster, Austria) for PRP sample preparations. PRP was prepared from ACDA whole blood by centrifuging it at 200 x g for 10 min and then further concentrated by an extra centrifugation step at 900 x g for 10 min to obtain the desired platelet concentration (3600 × 10^3^/μl). The sodium citrate anticoagulated plasma was used to prepare platelet poor plasma (PPP) by double centrifugation (2750 x g for 5 min and 10,000 x g for 10 min). Combining PRP and PPP, a series of different concentrations of validation samples were prepared with the platelet count between 250 and 3600 × 10^3^/μl.

### Instruments

The Sysmex® XN-9000 (Sysmex Corporation, Kobe, Japan) is a 5-part differential hematology analyzer, designed for high-throughput hematology laboratories. Platelet count determination is based on direct impedance with hydrodynamic focusing [14]. The analysis range for counting platelets in whole blood is between 0 and 5000 × 10^3^/μl.

The Sysmex® XP-300 (Sysmex Corporation, Kobe, Japan) is an automated 3-part differential hematology analyzer that was designed for measuring complete blood cell counts using the direct current detection method with coincidence correction. This device is considered as a POC, showing a great precision compared to other high-throughput hematology analyzers [15]. This device is suitable for measuring platelet count in the whole blood between 10 and 999 × 10^9^/L [16].

The PC100 platelet counter (Dutch Medical Devices, Valkenswaard, the Netherlands) is a POC device using a patented digital 3D image-based system to identify and count platelets [17]. Briefly, images of the blood sample are recorded through a glass slide covered by a plate. The mounted camera captures a unique optical pattern, indicative of platelet at a given location, which is then further processed by the software, resulting in the platelet count of the blood sample [17]. The device uses a unique disposable glass slide, containing two adjacent separate chambers which facilitates measurement of technical replicates. The platelet concentrations are determined at multiple locations to ensure high accuracy and measurement consistency. The platelet concentrations are determined at multiple locations. The analyzer requires 20 μl sample/measurement and provides the platelet count per nanoliter (plt/nL ) [12]. The results in plt/nL were converted into international units (× 10^3^/μl).

### Platelet count determination

EDTA anticoagulated whole blood was measured on Sysmex®XN-9000 and Sysmex® XP-300 according to the instruction manual. In short, undiluted whole blood was aspirated into the devices and platelet count was obtained. Whole blood platelet count was determined with the PC100 platelet counter after manual dilution (1:25) of the whole blood with ammonium oxalate solution (ThromboCount Pur; Bioanalytic GmbH, Umkirch/Freiburg, Germany). Platelet enumeration was performed in two technical replicates by using single glass slides with two adjacent chambers (chamber A and B).

Prior to platelet count determination in PRP, the validation samples were diluted to 250 × 10^3^/μl with homologous PPP and subsequently were measured by Sysmex® XP-300 in a single measurement. The PRP validation samples were diluted (1:100) with ThromboCount Pur and subsequently measured with PC100 platelet counters as described above for the whole blood samples.

### Statistical analysis

Statistical analysis was carried out by following the Clinical and Laboratory Standards Institute (CLSI) guideline for Measurement Procedure Comparison and Bias Estimation Using Patient Samples (EP09c) [18]. The closeness of agreement (trueness) between candidate method, the PC100 platelet counter and the reference methods, Sysmex® XN-9000 and Sysmex® XP-300 was assessed with Passing-Bablok linear regression and Bland-Altman analysis. Slope and intercept of Passing-Bablok regression were calculated with their 95% confidence interval (CI). Significant proportional or constant bias was considered when the 95% CIs of slope and intercept did not include 1 and 0, respectively. Furthermore, the clinical significance of differences between methods was determined by assessing the bias and its 95% CI calculated from the regression equation at medical decision points of 50, 100 and 600 × 10^3^/μl for whole blood or at extremes of the extremes of the measuring interval in PRP samples. The Bland-Altman’s method was applied to identify the mean of differences values between the candidate and reference methods and to visualize the underlying variability characteristics of relationship between the methods. Plotting the percentage difference values was preferred where the differences between methods were proportional to concentration of the reference methods. The equivalence between the methods is concluded when the bias was within a predefined acceptance criterion of ±10% or ± 10 × 10^3^/μl.

## Results

### Data control

All samples were independently quality controlled, and all measurements were performed within 3 h after blood collection to reduce the risk of preanalytical variation due to different time-intervals that may cause changes in platelet count [13, 19]. The platelet count obtained in the whole blood samples or in PRP was excluded upon the occurrence of any of the following criteria: i) either one of the reference methods or the PC100 platelet counter failed to obtain a value; ii) an assignable cause occurred; iii) the results were outside of the measuring interval. After data control and exclusion of non-eligible donors, platelet counts obtained from 102 and 60 donors were used for analysis of whole blood and PRP platelet counts, respectively.

### Platelet count in whole blood – method comparison

**Fig. 1:**
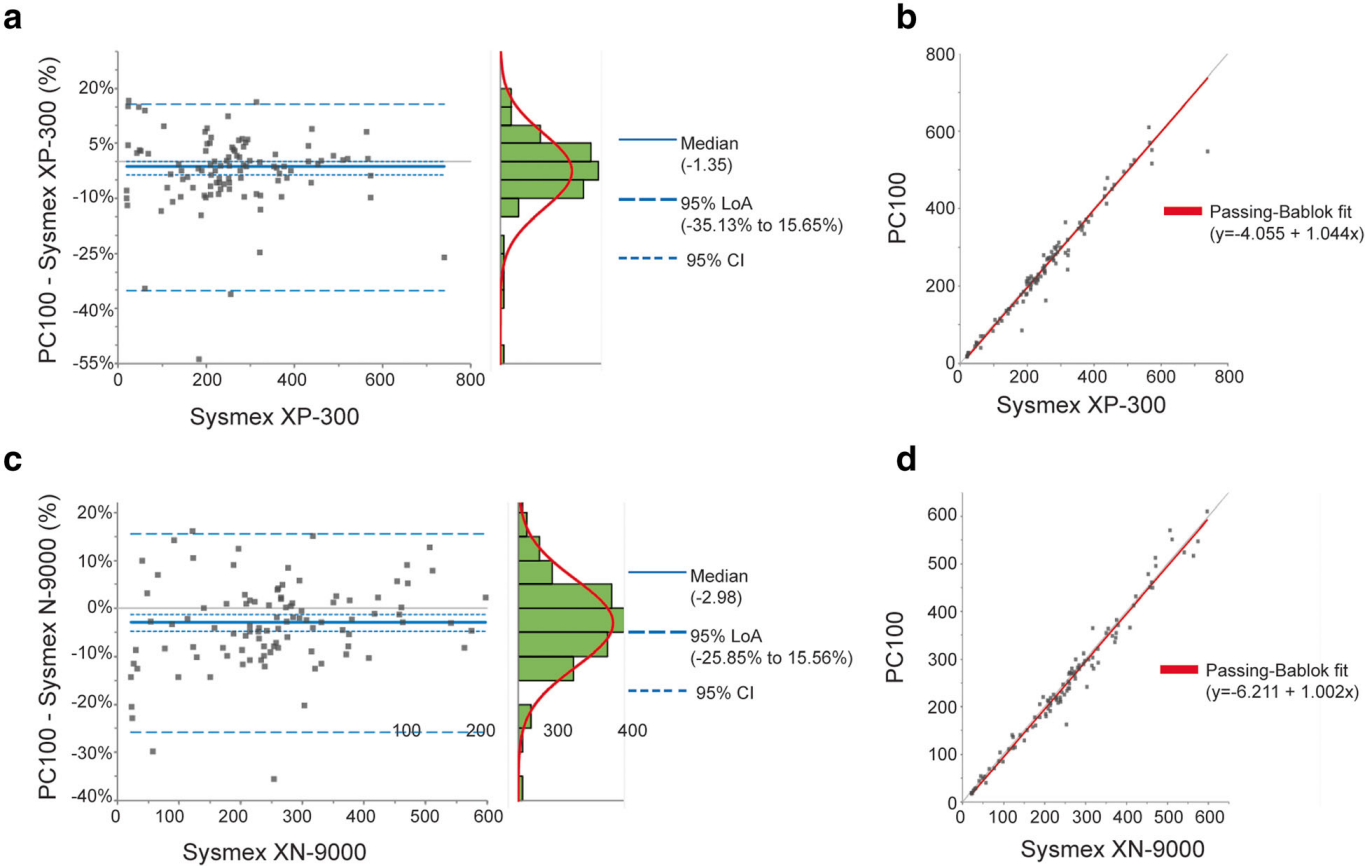
Differences between PC100 platelet counter and reference methods in measuring whole blood platelet count. The platelet count was determined using the PC100 platelet counter vs. the Sysmex® XP-300 (**a**,**b**) and the PC100 platelet counter vs. the Sysmex® XN-9000 (**c**,**d**). **a**,**c** Bland Altman plot visualizing the difference (%) between PC100 platelet counter and Sysmex® XP-300 or Sysmex® XN-9000. The grey lines represent the zero-difference line and the bold blue lines represents the median differences. **b**,**d** Passing-Bablok regression line is shown between the PC100 platelet counter and the reference methods. The grey line indicates the y = x line and the bold red line represents the regression line. *N* = 102

The direct comparison of PC100 platelet counters with the Sysmex® XP-300 and XN-9000 were performed using a Bland-Altman plot and Passing-Bablok regression method. The Bland-Altman difference plot revealed a heterogeneous distribution of data around the zero difference line with more than 95% of the points located within the 95% limits of agreement (LoA) (Fig. 1a,c). Given that Sysmex® XP-300 and XN-9000 are considered as representatives of the true value, the differences were plotted against the reference methods on the X axis. The median was chosen as an estimate of the bias for both comparisons due to the skewness in distribution of differences. The Passing-Bablok regression fit (Fig.1b,d) provided the estimate of slopes and intercept for both comparisons.

The median bias of the PC100 platelet counter against Sysmex® XP-300 and Sysmex® XN-9000 was − 1.35% (95%CI, − 3.56 to 0.12%) and − 2.98% (95%CI, − 4.82% to − 1.31%), respectively (Table 1.). A strong linear correlation (*r* ≥ 0.98) was found between the PC100 platelet counter and the reference methods, confirming the reliability of the regression results estimating a perfect slope of 1 for both comparisons.

**Table 1:** Results of the method comparison for in whole blood platelet count between the candidate method, PC100 platelet counter and reference methods, Sysmex® XP-300 and Sysmex® XN-9000

Parameter	***PC100 platelet counter*** vs. ***Sysmex® XP-300***		***PC100 platelet counter*** vs. ***Sysmex® XN-9000***	
	Estimate	95% CI	Estimate	95% CI
Median difference (%)	−1.35%	−3.56 to 0.12%	− 2.98%	−4.82% to − 1.31%
95% Lower LoA (%)	− 35.13%	–	− 25.85%	–
95% Upper LoA (%)	15.56%	–	15.56%	–
Slope	1.00	0.98 to 1.03	1.00	0.97 to 1.03
Intercept	−4.06	−11.38 to 1.24	− 6.21	− 14.59 to − 1.38
Correlation coefficient (r)	0.98	–	0.99	–
N_samples_	102	–	102	–
Measuring interval (×10^3^/μL)	19 to 739	–	21 to 597	–

*95% CI* 95% Confidence Interval, *Plts* Platelets, *LoA* Limit of Agreement

The 95% CI of slope was 0.98 to 1.03 (− 2 to 3%) and 0.97 to 1.03 (− 3 to 3%) when PC100 platelet counter was compared with the Sysmex® XP-300 or the Sysmex® XN-9000, respectively. In both comparisons, the predefined acceptance criterion (±10%) covered the 95% CI of proportional bias (slope) and median bias obtained from the Passing-Bablok algorithm and difference plot.

**Fig. 2:**
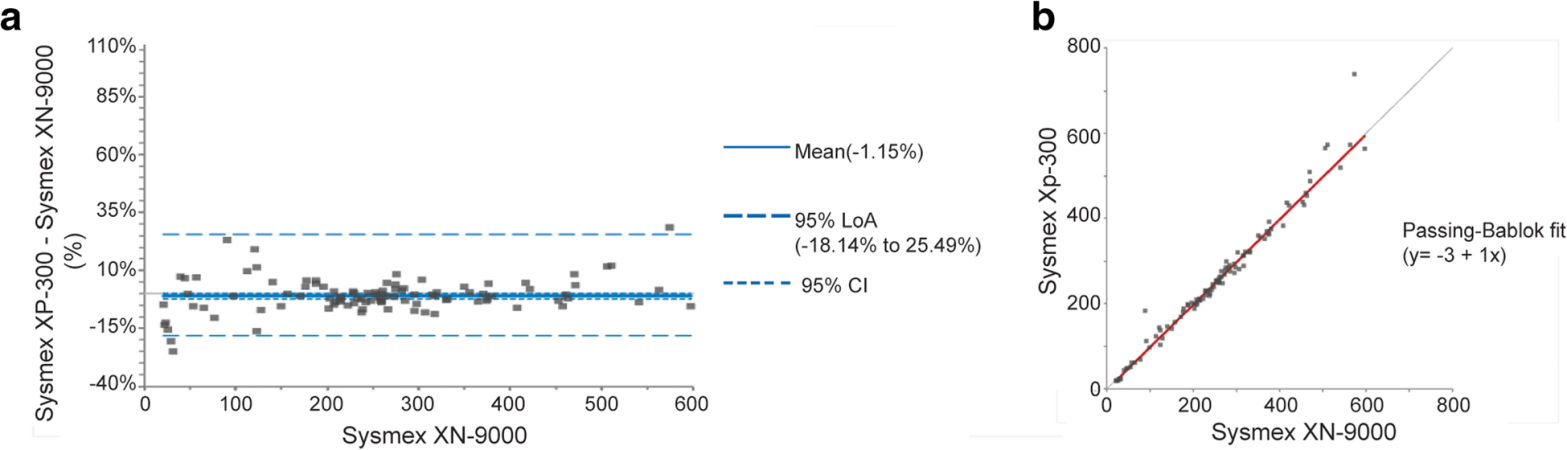
Method comparison for platelet count in whole blood between Sysmex® XP-300 and Sysmex® XN-9000. The platelet count was determined using the Sysmex® XP-300 or the Sysmex® XN-9000. **a** In the Bland Altman plot, the difference (%) between the Sysmex® XP-300 and the Sysmex®XN-9000 is plotted against the values obtained by Sysmex® XN-9000. Grey line represents the zero-difference line and the bold blue line represents the median difference. **b** In the Passing-Bablok regression plot, the grey line represents the y = x line and the bold red line represents the regression line. *N* = 102

Given the minor differences seen between the comparison of PC100 platelet counters against Sysmex® XP-300 and Sysmex® XN-9000, direct comparison of the two reference methods was conducted (Fig. 2, Table 2). To visualize the differences between the two methods, the Bland Altman plot was generated, showing the differences between the two methods against Sysmex® XN-9000 on the X axis (Fig. 2a). This plot indicated that difference values were heterogeneously scattered around zero difference line with more than 95% of the points falling within the 95% LoA. The median was chosen as the best estimate of central tendency due to presence of a potential outlier, leading to skewness in distribution of differences. The Passing-Bablok regression analysis was applied to provide an estimation of slope and intercepts (Fig. 2b).

**Table 2:** Results of the method comparison for platelet count in whole blood between Sysmex® XP-300 vs. Sysmex® XN-9000

Parameter	***Sysmex® XP-300*** vs. ***Sysmex® XN-9000***	
	Estimate	95% CI
Mean difference (× 10^3^/μL)	−1.15%	− 2.52 to 0.00%
95% Lower LoA (× 10^3^/μL)	− 18.14%	–
95% Upper LoA (× 10^3^/μL)	25.49%	–
Slope	1.00	0.97 to 1.02
Intercept	−3.00	−6.76 to 3.07
Correlation coefficient (r)	0.99	–
N_samples_	102	–
Measuring interval (×10^3^/μL)	21 to 597	–

*95% CI* 95% Confidence Interval, *Plts* Platelets, *LoA* Limit of Agreement

The median difference between two methods was − 1.15% (95% CI, − 2.25 to 0%). The relationship between the two reference methods was shown to be strongly linear (*r* = 0.99). The Passing-Bablok regression analysis revealed a slope of 1 with a 95%CI of 0.97 to 1.02 (− 3 to 2%) and an intercept of − 3 (95% CI, − 6.76 to 3.07) indicating the lack of either proportional or constant bias, and a perfect agreement between the two methods. The predefined acceptance criterion (±10%) covered the 95% CI of proportional bias (slope) and median bias obtained from the Passing-Bablok algorithm and difference plot, respectively.

### Platelet count in platelet rich plasma (PRP) – method comparison

In order to test the higher and the lower limits of the PC100 platelet counter, platelet count was measured in PRP validation samples consisting of 250–3600 × 10^3^/μl. Given the perfect agreement between the two reference methods validated using whole blood samples, platelet count in PRP was measured only with Sysmex® XP-300 for further analyses. The platelet count in PRP was measured using PC100 platelet counter and Sysmex® XP-300, and subsequently the differences were compared.

**Fig. 3:**
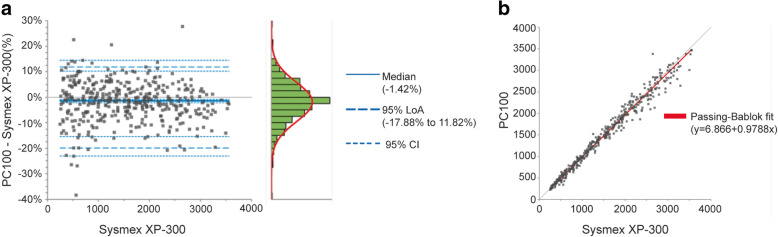
Differences between platelet count in PRP measured by PC100 platelet counter and Sysmex® XP-300. The platelet count in PRP was determined using PC100 platelet counter and Sysmex® XP-300. **a** Bland-Altman plot shows the differences between the two methods wherein the grey line represents the zero-difference line and the bold blue line shows the median difference. **b** Passing-Bablok regression analysis. *N* = 403

The direct comparison of PC100 platelet counter with the Sysmex® XP-300 was performed using Bland-Altman and Passing-Bablok regression analysis (Fig. 3.). The Bland-Altman plot showed heterogeneous distribution of data around the zero-difference line with more than 95% of the points being within the 95% LoA. The median difference between the PC100 platelet counter and Sysmex® XP-300 was − 1.42% (95% CI, 2.17 to 0.93%) (Fig. 3a, Table 3).

**Table 3:** Results of the method comparison for platelet count in PRP between the candidate method, PC100 platelet counter and the reference method, Sysmex® XP-300

Parameter	***PC100 platelet counter*** vs. ***Sysmex® XP-300***	
	Estimate	95% CI
Median difference (%)	−1.42%	−2.17% to − 0.93%
95% Lower LoA (%)	−19.88%	− 23.06% to − 15.43%
95% Upper LoA (%)	11.82%	10.17 to 14.39%
Slope	0.98	0.97 to 0.99
Intercept	6.87	−7.20 to 19.98
Correlation coefficient (r)	0.99	–
N_samples_	403	
Measuring interval (×10^3^/μL)	250 to 3556	–

*95% CI* 95% Confidence Interval, *Plts* Platelets, *LoA* Limit of Agreement

The Passing-Bablok algorithm was applied to estimate the slope and intercept, governing the linear relationship between the two methods (Fig. 3b.). It revealed that a slope of 0.98 (− 2%) with 95% CI of 0.97 to 0.99 (− 3 to 1%) (Table 3.). While 95% CI of the slope did not include 1, the 2% proportional bias and its 95% CI were within the 10% predefined acceptance criterion. Moreover, a correlation coefficient of 0.99 revealed a strong linear relationship between the two methods (Table 3).

### Clinical significance of bias

Following the estimated differences in platelet counts between the PC100 platelet counter and the reference methods, clinical significance of the bias was assessed in the whole blood and PRP samples. The clinical significance of bias was assessed in the whole blood samples at medical decision points of 50, 100 and 600 × 10^3^/μl. For the platelet counts measured in PRP samples, the clinical significance of bias was assessed at the extremes of the measurement interval (250–3600 × 10^3^/μl) since no reference interval or medical decision points have been reported for platelet counts in PRP.

The Passing-Bablok regression analysis was used to estimate the bias between the PC100 platelet counter and the reference methods at the predefined medical decision points (50, 100 and 600 × 10^3^/μl) (Table 4.). In the whole blood samples, the PC100 platelet counter showed the largest bias at the lowest medical decision point (50 × 10^3^/μl) regardless of the comparative method. Notably, the estimated bias in platelet count of whole blood was considerably reduced in both comparisons, as the platelet concentration was increased towards the higher medical decision points. Comparing with Sysmex® XP-300, the 95% CI of bias at all medical decision points was covered within the predefined acceptance criterion (±10% or ± 10 × 10^3^/μl). At all medical decision points, the predefined acceptance criterion (±10% or ± 10 × 10^3^/μl) covered the 95% CI of bias resulting from comparison of the PC100 platelet counter with Sysmex® XP-300. However, when compared to Sysmex® XN-9000, the predefined acceptance criterion only included the estimated 95% CI of bias at the highest medical decision point (600 × 10^3^/μl). Nevertheless, the estimated bias obtained from both comparisons were covered by the acceptance criterion (±10% or ± 10 × 10^3^/μl).

**Table 4:** Assessment of clinical significance of bias in platelet count of whole blood or PRP between the candidate method PC100 platelet counter and comparative reference methods, Sysmex® XP-300 and Sysmex® XN-9000

Sample type	***Compared methods***	***Reference method*** (X*(× 10^**3**^/μL))	***Candidate method*** (Y*(× 10^**3**^/μL))	***Bias*** (Y*-X*)	***95% CI***
Whole Blood	PC100 platelet counter vs. Sysmex XP-300	50	46.2	−3.85 (−7.7%)	−9.87 to 0.50 (−19.7% to −0.3%)
		100	96.4	−3.65 (− 3.6%)	−8.39 to − 0.28 (− 8.4% to − 0.3%)
		600	598.4	−1.62 (0.3%)	− 12.99 to 8.04 (−2.2% to −4.7%)
	PC100 platelet counter vs. Sysmex XN-9000	50	43.9	−6.13 (−12.3%)	−13.21 to −2.35 (26.4% to − 4.7%)
		100	93.9	−6.05 (− 6.1%)	− 11.82 to − 2.59 (− 11.8% to − 2.6%)
		600	594.7	−5.26 (− 0.9%)	−18.63 to 10.68 (− 3.1 to 1.8%)
PRP	PC100 platelet counter vs. Sysmex XP-300	250	251.6	1.56 (0.6%)	−10.32 to 12.32 (− 4.2 to 4.9%)
		3600	3432.5	− 67.46 (−1.9%)	− 99.93 to − 30.17 (− 2.9% to − 0.9%)

Regarding the PRP samples, the bias between the PC100 platelet counter and Sysmex® XP-300 were 1.56 (0.6%) and − 67.46 (− 1.9%) at the lower and upper limits of the measurement interval, respectively. The 95%CIs of bias were reasonably narrow considering the broad range of measuring interval and fell within the predefined acceptance criterion (10% or ± 10 × 10^3^/μl).

## Discussion

POC testing receives an increased attention in laboratory medicine as they could be a promising alternative for accelerating the diagnosis processes by shortening the turnaround times [20]. In this study, we compared the closeness of agreement (trueness) between two different methods for determining platelet counts in whole blood and PRP. We compared the results of a newly developed POC device, the PC100 platelet counter, with two reference hematology analyzers, Sysmex® XP-300 and Sysmex® XN-9000.

Our results revealed a strong equivalence between the PC100 platelet counter and reference methods for the defined applications. We found that for platelet counts in whole blood, the PC100 platelet counter showed a higher negative bias at lower counts and a tendency towards a closer agreement with comparative methods at increasing concentrations. For instance, the comparison against the Sysmex® XP-300 showed a bias of − 7.7% at 50 × 10^3^/μl which was reduced to − 0.3% at 600 × 10^3^/μl.

Regarding the platelet counts in PRP; however, the PC100 platelet counter demonstrated an overestimation of platelet counts at lower concentrations, followed by a progressive underestimation of platelet counts as the concentration increased. The comparison against the Sysmex® XP-300 revealed an absolute bias of 0.6% at 250 × 10^3^/μl which increased to 1.9% at 3600 × 10^3^/μl. These minor differences are likely attributed to the different algorithms used in the systems for particle detection and the classification of the detected particle as platelet or non-platelet.

The high correlation coefficient (*r* ≥ 0.98) shown in all method comparisons indicated a sufficient range of analyzed data considering dependency of correlation coefficient on the analytical range covered by the data [21]. However, the data points are not distributed evenly throughout the measuring interval with a larger percentage of samples collected from donors whose platelet count fell within the reference interval (150–400 × 10^3^/μl). Since the regression line is calculated by minimizing the distances of the data points to the regression line, one can argue that the regression equation will be dominantly governed by subintervals containing the majority of data. Such oversampling upon sample collection is often unavoidable given the scarcity of donors with very low or high concentrations.

Comparison of the PC100 platelet counter with Sysmex® XP-300 showed few notable aberrant data points which were found to be attributed to Sysmex® XP-300 considering its comparison to Sysmex® XN-9000. To address the nonparametric distribution of data, the Passing-Bablok regression was chosen to ensure robust regression estimates even in presence of outliers [22].

While our analyses showed minor analytical differences between the methods, the estimated bias of all differences fell within the acceptance criterion of ±10% or ± 10 × 10^3^/μl, concluding that the criteria for method equivalence had been met and the instruments can be used interchangeably for the defined applications.

## Conclusions

Taken together, the PC100 platelet counter provides a reliably fast method for platelet count determination. Given the capability of measuring platelet count in extremely high concentrations, the PC100 platelet counter can benefit the regenerative medicine efforts where PRP is used for various treatments [9, 10]. Furthermore, considering that changes in platelet count may serve as possible biomarkers in various diseases, the PC100 platelet counter may provide a valuable point-of-care device for accurate and fast results regarding platelet count.

## Data Availability

All data is available upon request.

## Abbreviations

POC: Point-of-care
PRP: Platelet Rich Plasma
PPP: Platelet Poor Plasma
EDTA: Ethylenediaminetetraacetic acid
ACDA: Acid citrate dextrose solution A
plt: Platelets
CI: Confidence interval
LoA: Limit of agreement
